# The clinicopathological and prognostic factors of hepatocellular carcinoma: a 10-year tertiary center experience in Egypt

**DOI:** 10.1186/s12957-022-02764-2

**Published:** 2022-09-19

**Authors:** Dina Sweed, Enas Sweed, Inas Moaz, Asmaa Mosbeh, Yahya Fayed, Sara Mohamed Abd Elhamed, Eman Sweed, Mahmoud Macshut, Shimaa Abdelsattar, Shimaa Kilany, Sara A. Saied, Reda Badr, Mahmoud S. Abdallah, Nermine Ehsan

**Affiliations:** 1 Pathology Department, National Liver Institute, Menoufia University, Shebin Elkom, Menoufia Egypt; 2 Radiology Department, Faculty of Medicine, Benha University, Benha, Egypt; 3 Epidemiology, and Preventive Medicine Department, National Liver Institute, Menoufia University, Shebin Elkom, Menoufia Egypt; 4 Hepatopancreatobiliary Surgery Department, National Liver Institute, Menoufia University, Shebin Elkom, Menoufia Egypt; 5 Clinical Pharmacology Department, Faculty of Medicine, Menoufia University, Shebin Elkom, Menoufia Egypt; 6 Clinical Biochemistry and Molecular Diagnostics Department, National Liver Institute, Menoufia University, Shebin Elkom, Menoufia Egypt; 7 Hepatology and Gastroenterology Department, National Liver Institute, Menoufia University, Shebin Elkom, Menoufia Egypt; 8 Clinical Pathology Department, National Liver Institute, Menoufia University, Shebin Elkom, Menoufia Egypt; 9 Clinical Pharmacy, Faculty of Pharmacy, University of Sadat City, Sadat City, Menoufia Egypt

**Keywords:** Hepatocellular carcinoma, Hepatitis C virus, Pathological subtypes, DAAs, prognosis

## Abstract

**Background:**

Hepatocellular carcinoma (HCC) remains a major health problem despite the emergence of several preventive and therapeutic modalities. HCC has heterogeneous and wide morpho-molecular patterns, resulting in unique clinical and prognostic criteria. Therefore, we aimed to study the clinical and pathological criteria of HCC to update the morpho-molecular classifications and provide a guide to the diagnosis of this disease.

**Methods:**

Five hundred thirty pathologically analyzed HCC cases were included in this study. The clinical and survival data of these cases were collected.

**Results:**

Hepatitis C virus is still the dominant cause of HCC in Egypt. Post-direct-acting antiviral agent HCC showed an aggressive course compared to interferon-related HCC. Old age, male gender, elevated alpha-fetoprotein level, tumor size, and background liver were important prognostic parameters. Special HCC variants have characteristic clinical, laboratory, radiological, prognostic, and survival data. Tumor-infiltrating lymphocytes rather than neutrophil-rich HCC have an excellent prognosis.

**Conclusions:**

HCC is a heterogenous tumor with diverse clinical, pathological, and prognostic parameters. Incorporating the clinicopathological profile per specific subtype is essential in the treatment decision of patients with HCC.

**Trial registration:**

This was a retrospective study that included 530 HCC cases eligible for analysis. The cases were obtained from the archives of the Pathology Department, during the period between January 2010 and December 2019. Clinical and survival data were collected from the patients’ medical records after approval by the institutional review board (IRB No. 246/2021) of Liver National Institute, Menoufia University. The research followed the guidelines outlined in the Declaration of Helsinki and registered on ClinicalTrials.gov (NCT05047146).

## Background

Hepatocellular carcinoma (HCC) is the sixth most common cancer worldwide and the third most common cause of cancer-related deaths [1]. In Egypt, HCC is the most common and the second most common cancer in males and females, representing 33.63% and 13.54% of all cancers, respectively [2, 3]. Hepatitis C virus (HCV) infection remains the most common cause of HCC in Egypt, even after the emergence of antiviral agents [3]. Unfortunately, there is no national surveillance program for HCC in Egypt yet [4]. HCC surveillance is a helpful tool for early detection, curative treatment, and better survival. The prognosis of HCC depends on various clinicopathological parameters, including serum alpha-fetoprotein (AFP) level, tumor size and focality, lymphovascular invasion (LVI), pathological stage and grade, and the background liver [5, 6]. Moreover, studies showed that achieving sustained virological response (SVR) has not decreased the risk of HCC post-direct-acting antiviral agents (DAAs) [7]. DAAs have been claimed to impact the clinical and histopathological parameters of HCC more than did interferon (IFN) therapy [7].

Treatment modalities for HCC have been improved dramatically over the last decades. However, the precise targeted agent per patient, improving the response, and overcoming drug resistance should be focus of ongoing HCC studies [8]. Integrating clinical and patient performance data, tumor overload, the status of background liver, and the morpho-molecular criteria of HCC could provide algorithms for tumor eradication either removal by surgery or transplantation or destruction by Sorafenib, chemo-embolization, radiofrequency ablation, and immunotherapy [9].

HCC has heterogeneous and wide morphological patterns, which have been attempted to identify to determine the specific molecular profiles and characteristics of each morphological subtype [10]. As a side note, there is a 10-year gap between the recent and the previous World Health Organization (WHO) classifications of tumors of the digestive system [11, 12]. With the emergence of new subtypes, a modification in the morphological subtypes of HCC has been proposed, and a three-tiered grading system has been endorsed in the fifth edition of WHO classification [12]. There was also an update in the eighth edition of the American Joint Committee of Cancer (AJCC) on the pathological staging of HCC [13]. Incorporating the clinicopathological profile per specific subtype is essential in the treatment decision of patients with HCC.

This study aims to illustrate the status of HCC in Egypt regarding the clinicopathological features, the impact of different HCV treatment modalities, and the prognostic issues. In addition, this study could provide descriptive data for the recently added HCC subtypes with focusing on their prognostic behavior. Understanding these issues could provide a comparative analysis of HCC in different countries and help to customize efforts in the surveillance program for HCC prevention and treatment.

## Methods

### Design and subjects

This was a retrospective study that included 530 HCC cases eligible for pathological analysis. The cases were obtained from the archives of the Pathology Department, during the period between January 2010 and December 2019. The collected data included the patients’ age, gender, virology status, prior viral treatment, and serum AFP level. All cases were reevaluated by three liver histopathologists according to the fifth edition of the WHO classification of digestive tumors and the eighth edition of the AJCC staging system. The pathological data included tumor size and focality, tumor pathological grade, pattern, clear/fatty cell changes, Tumor infiltrating lymphocytes (TILs), the presence of intra-tumoral fibrosis, pathognomonic bile formation, necrosis, LVI, bile duct and perineural invasion, pathological stage, and Lymph node (LN) status [12, 13].

Regular follow-up was conducted either in the oncology clinic or through personal contact every 3 months during the first year, 6 months during the second and third years, and then yearly thereafter. Tumor recurrence was defined as the occurrence of a tumor after a period of remission during which initial cancer could not be detected.

Overall survival (OS) was calculated from the date of diagnosis to the time of death or the last follow-up visit.

#### Inclusion criteria

Any HCC cases attempted pathological evaluation in the last 10 years including primary HCC cases and metastatic cases (before the primary site was discovered. Liver tissue specimens were obtained either by needle biopsy to establish the diagnosis in atypical radiological cases, after surgical resection, or liver transplantation.

#### Exclusion criteria

HCC developed in pediatric patients. Any HCC patients who received neo-adjuvant chemotherapy, radiofrequency ablation, microwave ablation, or Sorafenib prior to surgery.

### Statistical analysis

Data were analyzed using the IBM SPSS software package version 20.0. (IBM Corp., Armonk, NY, USA). The Kolmogorov–Smirnov test was used to verify the normality of the distribution of variables. Comparisons between groups for categorical variables were assessed using the Chi-square test (Fisher or Monte Carlo). The Student *t* test was used to compare two groups for normally distributed quantitative variables, while the analysis of variance was used to compare the groups. The Mann–Whitney *U* test was used to compare two groups for abnormally distributed quantitative variables, while the Kruskal–Wallis test was used to compare different groups for abnormally distributed quantitative variables. The Kaplan–Meier plots and log-rank test were used to evaluate the patients’ OS. The significance of the obtained results was judged at the 5% level.

### Ethical considerations

Clinical and survival data were collected from the patients’ medical records after approval by the institutional review board (IRB No. 246/2021) of Liver National Institute, Menoufia University. The research followed the guidelines outlined in the Declaration of Helsinki and registered on ClinicalTrials.gov (NCT05047146).

## Results

These 530 HCC cases were divided into 513 primary HCC cases and 17 metastatic cases. The sites of metastasis were the bone (4 cases), suprarenal glands (4 cases), lung (3 cases), LNs (2 cases), and duodenum, epigastrium, brain, and skin (1 case each). Liver tissue specimens were obtained either by needle biopsy (145 cases), after surgical resection (319 cases), or after liver transplantation (49 cases).

**Fig. 1:**
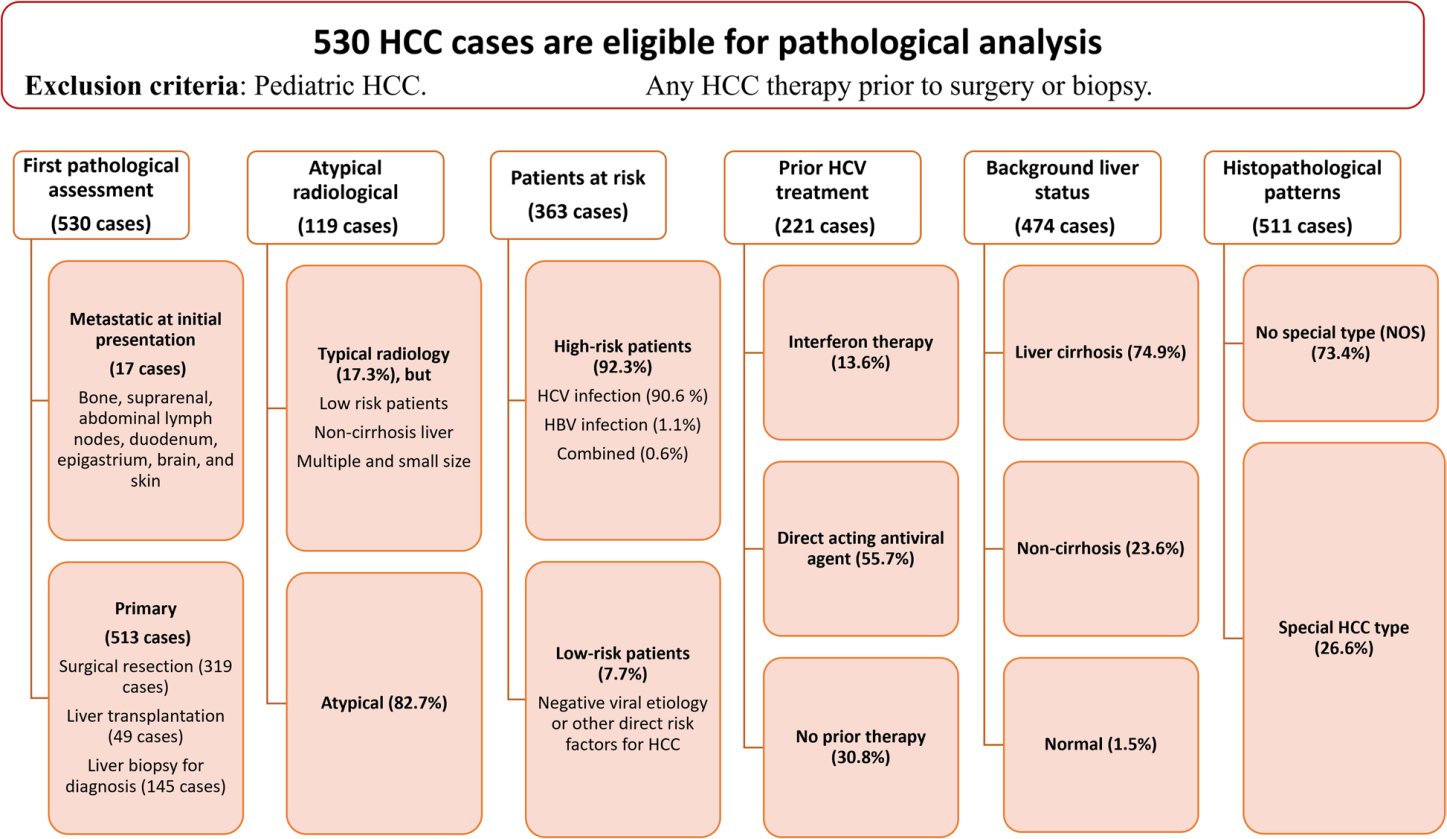
The primary parameters used to conduct comparative analyses

Figure 1 depicted the primary parameters that we used to conduct comparative analyses

### General demographic data of primary HCC cases

The median age of the patients was 58 years (range, 13–82 years), and the patients were predominantly male, with a male/female ratio of 5.16:1. In all patients, hepatitis was the most common cause of HCC: chronic HCV in 329/363 (90.6%) patients, hepatitis B virus (HBV) in 4/363 (1.1%) patients, and combined HCV and HBV in 2 (0.6%) patients. An associated bilharzial infection was reported in only six patients (1.2%). The median serum AFP level was 29 ng/dL, with 60% of patients having a serum AFP level of ≥ 20 ng/dL, as shown in Table 1.

**Table 1:** General clinical, laboratory, and pathological data of HCC

Parameters		***n*** (%)
**Clinical and laboratory**	**Median age (years) (*n*= 511)**	58 years
	< 60	287 (57.1)
	≥ 60	216 (42.9)
	**Gender (*n*= 511)**	
	Male	428 (83.8)
	Female	83 (16.2)
	**Etiology (*n*= 363)**	
	HCV	329 (90.6)
	HBV	4 (1.1)
	Combined HCV/HBV	2 (0.6)
	Non-viral	28 (7.7)
	**Previous HCV treatment (*n*= 221)**	
	IFN	30 (13.6)
	DAAs	123 (55.7)
	No	68 (30.8)
	**Median AFP (*n*= 300)**	29 ng/dl
	AFP (≥ 20 ng/dl)	180 (60)
	AFP (≥ 200 ng/dl)	81 (27)
	AFP (≥ 400 ng/dl)	52 (17.3)
**Macroscopic**	**Tumor focality (*n*= 492)**	
	Solitary	333 (67.7)
	Multiple	159 (32.3)
	**Median tumor size (*n*= 480)**	5 cm
	Tumor size ≥ 2 cm	430 (89.6)
	Tumor size ≥ 5 cm	255 (53.1)
**Microscopic**	**Pathological grade (*n*= 511)**	
	I	34 (6.6)
	II	357 (69.9)
	III	82 (16)
	Un	38 (7.5)
	**Pathological patterns of NOS (*n*= 375)**	
	Trabecular and acinar	341 (90.9)
	Solid	34 (9.1)
	**Tumor clear and fatty cell changes (*n*= 511)**	
	Absent	288 (56.4)
	Present	223 (43.6)
	**TILs Salgado classifications (*n*= 511)**	
	0–10	459 (89.8)
	20–40	51 (10.0)
	50–90	1 (0.2)
	**Intra-tumoral fibrous stroma (*n*= 511)**	
	Absent	344 (67.3)
	Present	167 (32.7)
	**Tumor necrosis (*n*= 511)**	
	Negative	360 (70.5)
	Positive	151 (29.5)
	**Pathological stage (*n*= 368)**	
	1a	24 (6.5)
	1b	121 (32.9)
	2	161 (43.8)
	3	49 (13.3)
	4	13 (3.5)
	**LVI (*n*= 372)**	
	Negative	201 (54.0)
	Positive	171 (46.0)
	**Bile duct invasion (*n*= 368)**	
	Negative	365 (99.2)
	Positive	3 (0.8)
	**Perineural invasion (*n*= 368)**	
	Negative	365 (99.2)
	Positive	3 (0.8)
	**LN status (*n*= 39)**	
	Negative	29 (74.4)
	Positive	10 (25.6)

*HCC* hepatocellular carcinoma, *HCV* hepatitis C virus, *HBV* hepatitis B virus, *IFN* interferon, *DAAs* direct acting anti-viral, *AFP* alpha-fetoprotein, *TILs* tumor-infiltrating lymphocytes, *LVI* lymphovascular invasion, *LN* lymph node. *Data were missed in some cases; we figured out the number beside each parameter*

Six cases were reported to have had a history of another malignancy before HCC diagnosis: two cases of prostatic carcinoma and one case each of urothelial carcinoma, colorectal carcinoma, papillary renal cell carcinoma, and lymphoma.

### Pathological data of primary HCC cases

**Fig. 2:**
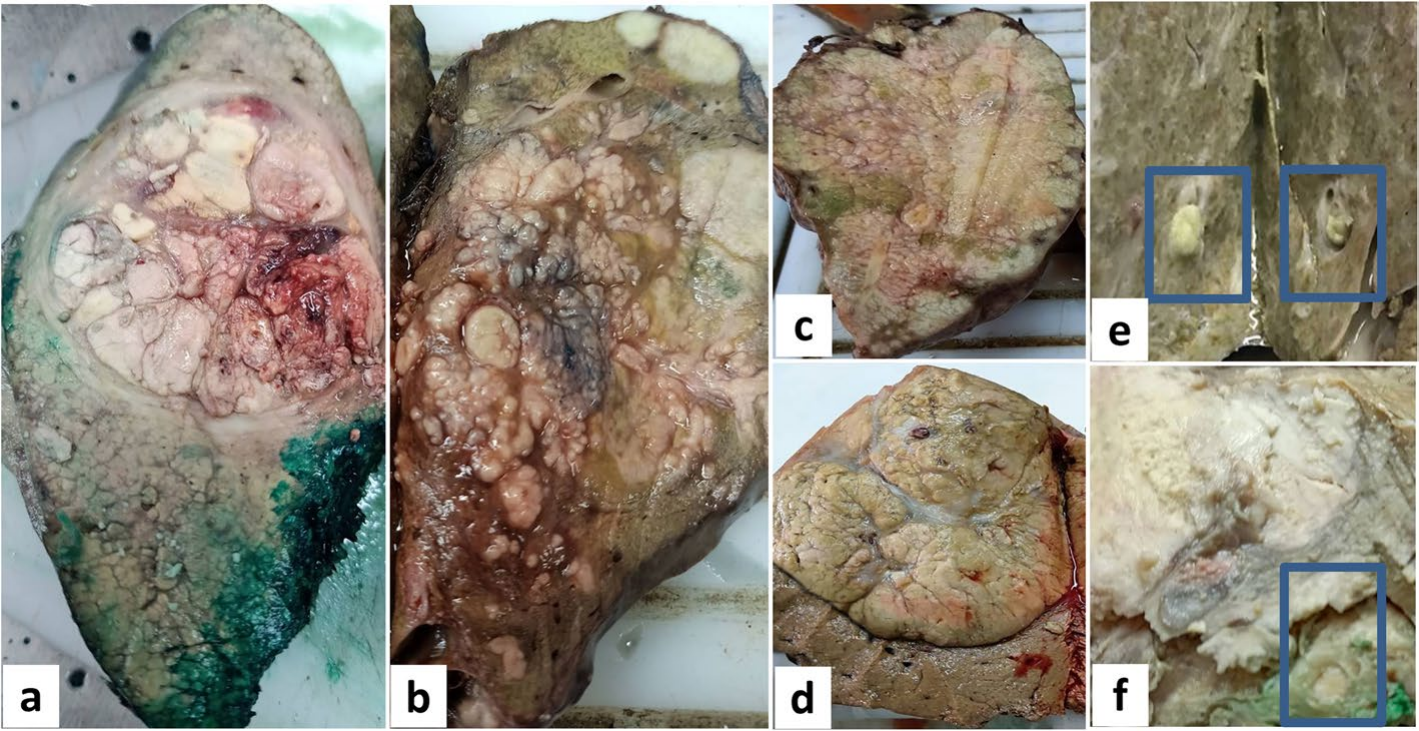
Different macroscopic appearance of HCC. **a** Solitary pseudocapsulated nodule in cirrhotic liver. **b** A diffuse or cirrhometric HCC with multiple satellite nodules in non-cirrhotic liver. **c** An infiltrative HCC. **d** A solitary pseudocapsulated HCC; however, in non-cirrhotic liver. **e** A macroscopic bile duct invasion in HCC cases (blue boxes). **f** A macroscopic lymphovascular invasion in HCC case (blue box)

The gross picture of HCC varied between pseudo-capsulated, circumscribed, multicentric with satellite nodules, and infiltrative. The color ranged from white to yellow, to green with an area of hemorrhage and necrosis. The background liver showed cirrhotic or non-cirrhotic changes (Fig. 2).

The majority of HCC cases (73.4%) were of the classic type, and “not otherwise specified” with pathognomonic bile was seen in 24.5% of cases. LVI, bile duct invasion, and perineural invasion were reported in 46%, 0.8%, and 0.8% of cases, respectively (Table 1).

**Fig. 3:**
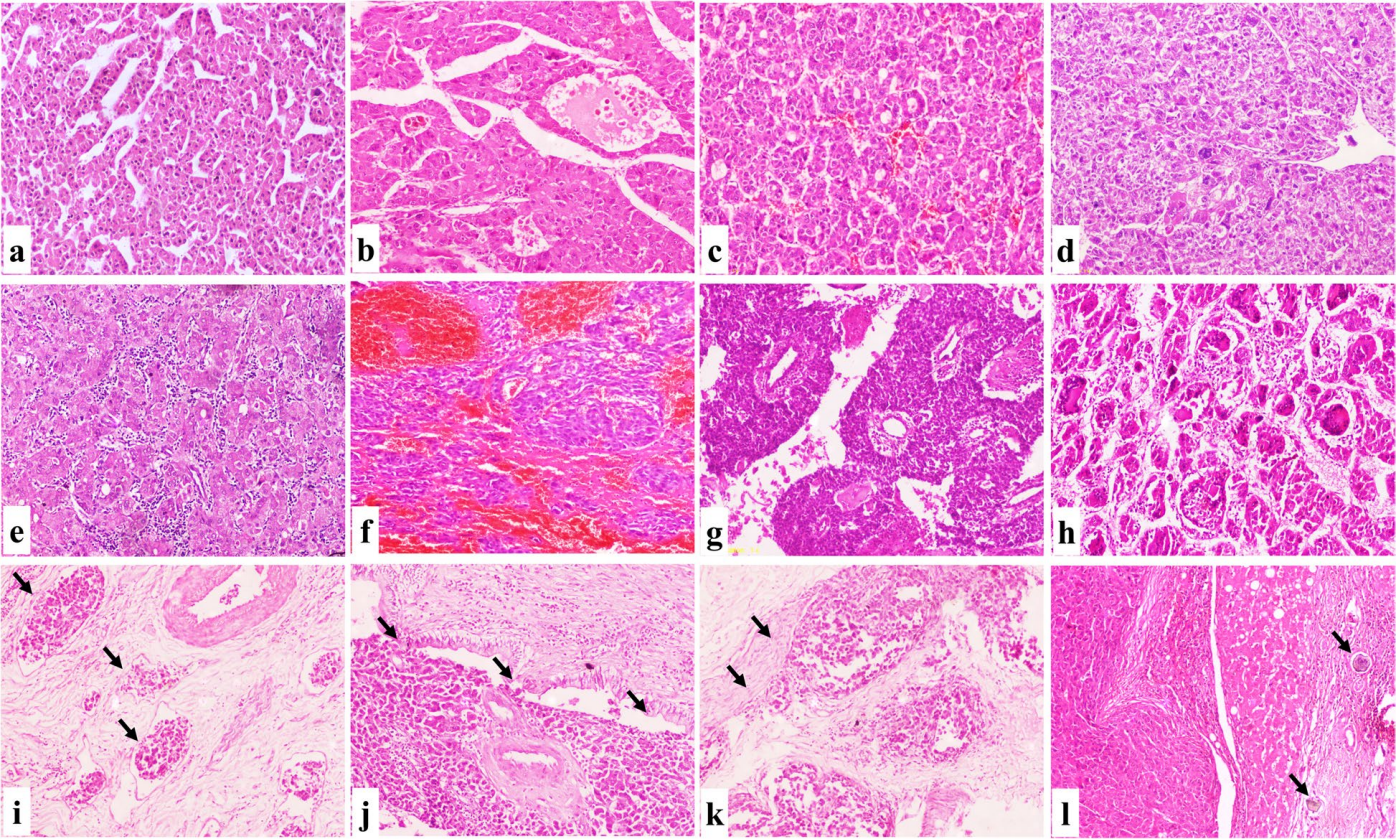
Microscopic aspects of HCC, NOS. **a** A well differentiated HCC showed thin trabaecule (IHC, 100×). **b** A moderately differentiated HCC showed wide acini filled with eosinophilic to bile secretion (IHC, 100×). **c** A moderately differentiated HCC showed mixed acinar and trabecular pattern (IHC, 100×). **d** A poorly differentiated HCC showed solid pattern with marked nuclear atypia (IHC, 100×). **e** HCC, NOS with mild intra-tumoral lymphocytes (IHC, 100×). **f** HCC, NOS with hemangiopericytoma like pattern (IHC, 100×). **g** HCC, NOS with prominent osteoclast like giant cells (IHC, 100×). **h** HCC with prominent lymphovascular invasion (arrows) (IHC, 100×). **i** HCC with bile duct invasion with attached tumor emboli to the epithelial cells (arrows) (IHC, 100×). **j** HCC with perineural invasion (arrows) (IHC, 100×). **k** HCC, NOS associated with calcified bilharzial ova (arrows) (IHC, 100×)

The background liver was normal, had chronic hepatitis, and had cirrhosis in 1.5%, 23.6%, and 74.9% of cases, respectively. Inflammatory activity was graded as mild, moderate, and marked in 45.9%, 50.8%, and 3.3% of cases, respectively. The presence of associated steatosis was encountered in 22.9% of cases (Fig. 3).

### Clinical factors affecting HCV-related HCC

Regarding age, a significant association was found only between a young age (< 40 years) and a high serum AFP level (*p =* 0.034). Regarding gender, being male was significantly associated with tumor multifocality and late pathological stage compared with being female (*p* = 0.013 and *p* = 0.021, respectively). In addition, intra-tumoral fibrosis was significantly observed in males (*p =* 0.001).

We also studied the impact of serum AFP levels on the clinicopathological parameters. The cases were allocated into three subgroups based on the serum AFP level (< 20, ≥ 20–400, and ≥ 400 ng/dL). Higher serum AFP levels were significantly associated with a younger age (*p* = 0.032). Serum AFP levels tended to be lower in post-DAA HCC cases followed by post-IFN cases compared with cases with no prior treatment (*p =* 0.069). Serum AFP levels of > 400 ng/mL was significantly associated with a large tumor size, advanced tumor pathological stage, and high tumor recurrence (*p* < 0.001, *p =* 0.033, and *p* = 0.026, respectively).

Data regarding the effect of prior HCV treatment was available from 221 cases. Among these cases, 13.6%, 55.7%, and 30.8% had post-IFN HCC, post-DAA HCC, and not undergone treatment, respectively. Post-IFN HCC was significantly associated with a unifocal tumor (*p* = 0.046). Furthermore, tumor necrosis was significantly higher in post-DAA HCC cases compared with the other groups (*p* = 0.02) (Table 2).

**Table 2:** The impact of HCV treatment modalities on HCC

Parameters	Post-IFN ***n*** = 30	Post-DAAs ***n*** = 123	No treatment ***n*** = 68	***p*** value
**Age (*n*= 221)**				
Mean ± SD	59.23 ± 6.44	57.25 ± 8.33	58.73 ± 9.75	0.2
Median	59.00	57.00	60.00	
**Gender (*n*= 221)**				
Male	24 (80.0%)	103 (83.7%)	54 (79.4%)	0.727
Female	6 (20.0%)	20 (16.3%)	14 (20.6%)	
**AFP (*n*= 162)**				
< 200 ng/dl	18 (66.7%)	70 (79.5%)	34 (72.3%)	0.340
≥ 200 ng/dl	9 (33.3%)	18 (20.5%)	13 (27.7%)	
**Tumor focality (*n*= 220)**				
Solitary	27 (90.0%)	83 (68.0%)	46 (67.6%)	0.046
Multiple	3 (10.0%)	39 (32.0%)	22 (32.4%)	
**Tumor size (*n*= 221)**				
Mean ± SD	5.89 ± 3.30	5.59 ± 3.53	5.26 ± 2.89	0.63
Median	4.50	4.00	4.50	
**Pathological grade (*n*= 203)**				
I	1 (3.6%)	10 (8.8%)	4 (6.6%)	0.779
II	20 (71.4%)	83 (72.8%)	47 (77.0%)	
III	7 (25.0%)	21 (18.4%)	10 (16.4%)	
**Tumor necrosis (*n*= 221)**				
Mean ± SD	8.50 ± 17.96	12.56 ± 22.81	3.82 ± 10.72	0.02
**Pathological stage (*n*= 173)**				
Early stage	24 (92.3%)	76 (81.7%)	45 (83.3%)	0.173
Late stage	2 (7.7%)	17 (18.3%)	9 (16.7%)	
**LVI (*n*= 177)**				
Negative	16 (59.3%)	50 (52.1%)	25 (46.3%)	0.536
Positive	11 (40.7%)	46 (47.9%)	29 (53.7%)	
**Non-tumor liver (*n*= 217)**				
Cirrhosis	23 (79.3%)	86 (70.5%)	40 (60.6%)	0.157
Non-cirrhosis	6 (20.7%)	36 (29.5%)	26 (39.4%)	
**Non-tumor inflammatory activity (*n*= 171)**				
Mild	12 (44.4%)	38 (42.7%)	29 (52.7%)	0.796
Moderate	14 (51.9%)	47 (52.8%)	23 (41.8%)	
Marked	1 (3.7%)	4 (4.5%	3 (5.5%)	

*HCC* hepatocellular carcinoma, *HCV* hepatitis C virus, *IFN* interferon, *DAAs* direct acting anti-viral, *AFP* alpha-fetoprotein, *LVI* lymphovascular invasion. *Data were missed in some cases; we figured out the number beside each parameter*

### Impact of etiology and background liver on the clinicopathological parameters of HCC

Patients without a viral etiology developed HCC at a younger age compared with those with a viral etiology (*p* = 0.002). The fibrolamellar carcinoma (FLC) variant represented nearly 25% of non-viral-related HCC and was significantly occurred on a normal liver background (*p* < 0.001).

The impact of background liver regardless of the etiological cause was studied. Non-cirrhotic HCC significantly occurred in the older age group (*p* = 0.05). In addition, a non-cirrhotic liver was significantly associated with tumor multifocality and large size (*p* = 0.01 and *p* < 0.001, respectively). LVI and bile duct invasion were significantly found in HCC raising on top of non-cirrhotic liver (*p* = 0.02, for both). A solid HCC pattern was associated with the non-cirrhotic liver background (*p* = 0.01) (Table 3).

**Table 3:** The clinicopathological criteria of HCC on top of cirrhotic and non-cirrhotic liver

Parameters	Cirrhosis ***n*** = 355	Non-cirrhosis ***n*** = 119	***p*** value
**Age (*n*= 474)**			
Mean age ± SD	57.21 ± 9.02	58.51 ± 10.43	0.05
Median	58.00	60.00	
**Gender (*n*= 474)**			
Male	292 (82.3%)	104 (87.4%)	0.19
Female	63 (17.7%)	15 (12.6%)	
**Etiology (*n*= 346)**			
HCV	229 (92.0%)	90 (92.8%)	1.00*
HBV	3 (1.2%)	1 (1.0%)	
Combined HCV/HBV	2 (0.8%)	0 (0.0%)	
Non-viral	15 (6.0%)	6 (6.2%)	
**Previous HCV treatment (*n*= 217)**			
IFN	23 (15.4%)	6 (8.8%)	0.16
DAAs	86 (57.7%)	36 (52.9%)	
No treatment	40 (26.8%)	26 (38.2%)	
**Serum AFP (*n*= 258)**			
< 200 ng/ml	139 (74.3%)	52 (73.2%)	0.85
≥ 200 ng/ml	48 (25.7%)	19 (26.8%)	
**Tumor focality (*n*= 350)**			
Solitary	247 (93.6%)	73 (84.9%)	0.01
Multiple	17 (6.4%)	13 (15.1%)	
**Tumor size (*n*= 350)**			
Mean ± SD	5.51 ± 3.68	6.59 ± 3.56	< 0.001
**Pathological grade (*n*= 443)**			
I	28 (8.5%)	5 (4.5%)	0.15
II	253 (76.4%)	83 (74.1%)	
III	50 (15.1%)	24 (21.4%)	
**Pathological patterns of NOS (*n*= 350)**			
Trabecular and acinar	247 (93.6%)	73 (84.9%)	0.01
Solid	17 (6.4%)	13 (15.1%)	
**Pathological stage (*n*= 355)**			
Early stage	232 (84.4%)	61 (76.2%)	0.09
Late stage	43 (15.6%)	19 (23.8%)	
**LVI (*n*= 358)**			
Negative	157 (57.1%)	35 (42.2%)	0.02
Positive	118 (42.9%)	48 (57.8%)	
**Bile duct invasion (*n*= 360)**			
Negative	275 (100%)	82 (96.5%)	0.012
Positive	0 (0%)	3 (3.5%)	

*HCC* hepatocellular carcinoma, *HCV* hepatitis C virus, *HBV* hepatitis B virus, *IFN* interferon, *DAAs* direct acting anti-viral, *AFP* alpha-fetoprotein, *LVI* lymphovascular invasion. *Data were missed in some cases; we figured out the number beside each parameter*

### Role of radiological imaging in diagnosing HCC and factors that interfere with the radiological findings

**Fig. 4:**
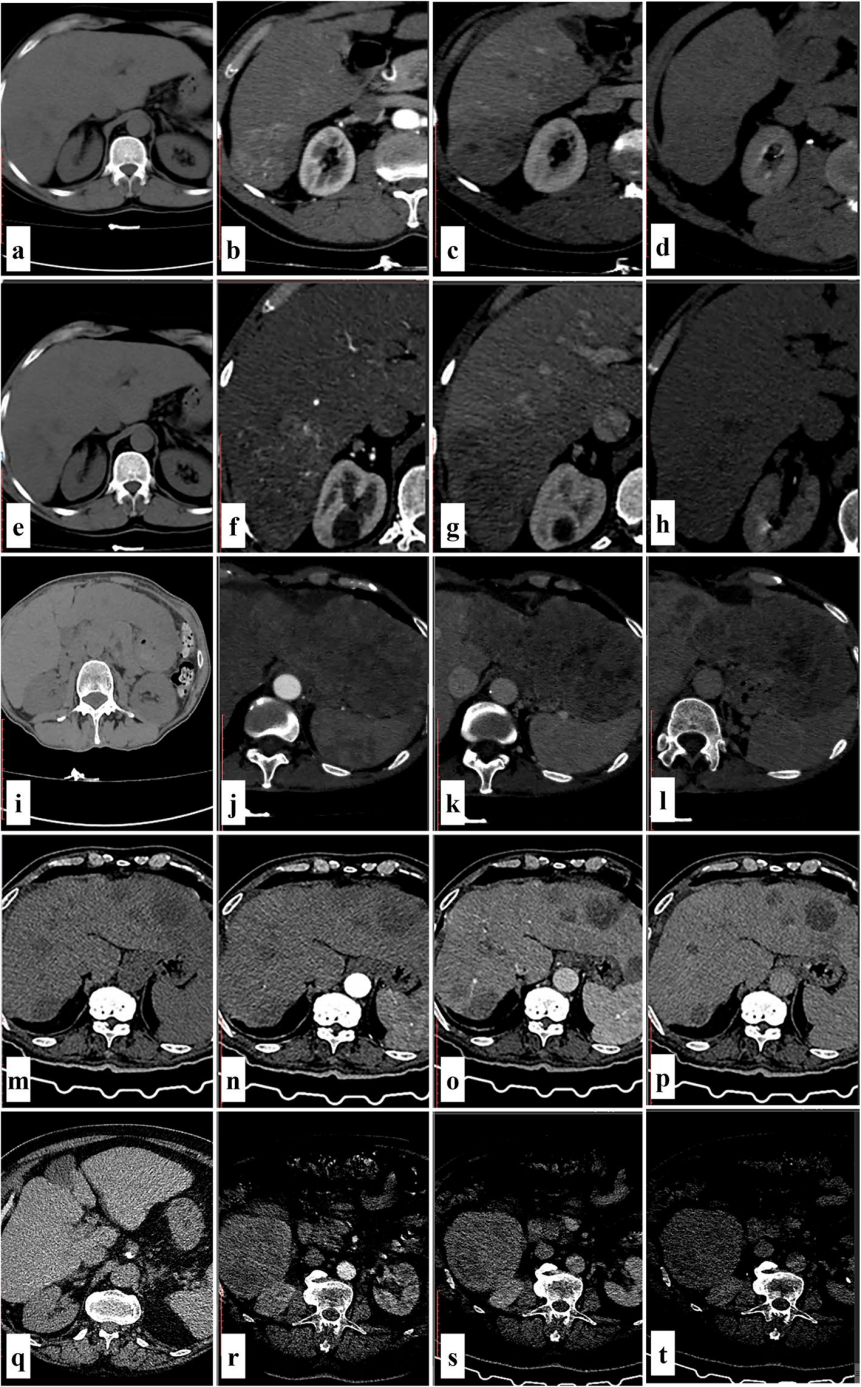
Contrast-enhanced triphasic CT imaging of typical and atypical HCC cases: A case of typical radiological appearance of multiple HCC (LR-5) (**a**–**d**). **a** Cirrhotic liver changes, **b** with right hepatic lobe focal lesion seen at segment VI displaying intense arterial enhancement, **c** with washout of contrast in portovenous phase, **d** being hypo dense to hepatic parenchyma in delayed equilibrium study. **e**–**h** Another focal lesion is seen at segment VII with similar enhancement pattern. A case of infiltrative HCC (LR-5) (**i**–**l**). **i** Cirrhotic liver changes, **j** with malignant infiltration of the left hepatic lobe that shows heterogeneous enhancement in the arterial phase with low density areas indicative of necrosis **k** and displays wash out of contrast at portovenous phase, **l** being hypodense to hepatic parenchyma in delayed equilibrium study. A case of multiple hypovascular HCC (LR-5) (**m**–**p**). **m** Cirrhotic liver changes, **n**–**p** with multiple bilobar variable sized hepatic focal lesions showing no contrast uptake in different study phases, the largest at left hepatic lobe segment II measuring 5 × 4.8 cm. A case of HCC on top of non-cirrhotic liver (LR-M) (**q**–**t**). **q** Non cirrhotic liver, **r** with right hepatic lobe segment VI large exophytic well defined focal mass lesion displaying thick irregular peripheral arterial enhancement and central hypo dense area of necrosis, **s** with washout of contrast at portovenous phase, **t** and delayed phases

Contrast-enhanced triphasic computed tomography (CE-CT) and dynamic magnetic resonance imaging (MRI) are the main methods used in radiological imaging, and both are crucial in the diagnosis of HCC. However, 145 HCC cases got liver biopsy as part of this investigation for two main reasons, Fig. 4. The first is typical radiological findings in low-risk patients, such as multiple lesions (17.3%) or low serum AFP levels. The second is abnormal radiological findings that warrant a liver biopsy and pathology (82.7%). Among these characteristics were hypovascular (40%), heterogeneous enhancement with no washout until the delayed phases (22%), huge infiltrative (18%), targetoid appearance (9%), and intra-ductal growth patterns (5%). Less likely features included multilocular cystic lesions (3%), central scars (2%), and capsular retraction (1%). In Table 4, the radiological characteristics of atypical HCC were discussed.

**Table 4:** The radiological characteristics of atypical HCC cases

	No wash-out until delayed phase ***n*** = 22	Huge infiltrative ***n*** = 18	Hypovascular ***n*** = 40	Intra-ductal growth ***n*** = 5	Targetoid ***n*** = 9	Typical ***n*** = 19	***p*** value
**Age (*n*= 111)**							0.349
Less	12 (54.5)	9 (50)	11 (28.9)	2 (40)	5 (55.6)	7 (36.8)	
More	10 (45.5)	9 (50)	27 (71.1)	3 (60)	4 (44.4)	12 (63.2)	
**Patients at risk (*n*= 69)**							0.874
Low risk	5 (27.8)	2 (20)	3 (15.8)	0 (0)	1 (14.3)	1 (10)	
High risk	13 (72.7)	8 (80)	16 (84.2)	4 (100)	7 (85.7)	9 (90)	
**Serum AFP (*n*= 76)**							0.047
Low	9 (64.3)	13 (86.7)	18 (64.3)	1 (25.0)	4 (66.7)	9 (100)	
High	5 (35.7)	2 (13.3)	10 (35.7)	3 (75.0)	2 (33.3)	0 (0)	
**Tumor size (*n*= 98)**							0.001
Less	3 (15.8)	0 (0)	2 (5.9)	0 (0)	0 (0)	13 (72.2)	
More	16 (84.2)	15 (100)	32 (94.1)	5 (100)	7 (100)	5 (27.8)	
**Focality (*n*= 113)**							0.037
Solitary	13 (59.1)	12 (66.7)	30 (75.0)	3 (60.0)	4 (44.4)	6 (31.6)	
Multiple	9 (40.9)	6 (33.3)	10 (25.0)	2 (40.0)	5 (55.6)	13 (68.4)	
**Tumor grade (*n*= 113)**							0.079
I	2 (9.1)	0 (0)	0 (0)	0 (0)	1 (11.1)	1 (5.3)	
II	14 (63.6)	14 (77.8)	31 (77.5)	3 (60.0)	4 (44.4)	15 (78.9)	
III	6 (27.3)	4 (22.2)	8 (20.0)	1 (20.0)	4 (44.4)	1 (5.3)	
Un	0 (0)	0 (0)	1 (2.5)	1 (20.0)	0 (0)	2 (10.5)	
**Histopathological pattern (*n*= 111)**							0.394
NOS	20 (90.9)	12 (66.7)	31 (77.5)	1 (20.0)	3 (33.3)	2 (10.5)	
Clear	2 (9.1)	4 (22.2)	7 (17.5)	2 (80.0)	5 (55.6)	16 (84.2)	
Scirrhous	0 (0)	2 (11.1)	2 (5.0)	0 (0)	1 (11.1)	0 (0)	
Steatohepatitis	0 (0)	0 (0)	0 (0)	0 (0)	0 (0)	1 (5.3)	
**Liver background (*n*= 110)**							0.223
Non-cirrhosis	7 (33.3)	2 (11.1)	12 (30.8)	3 (60.0)	3 (37.5)	8 (42.1)	
Cirrhosis	14 (66.7)	16 (88.9)	27 (69.2)	2 (40.0)	5 (62.5)	11 (57.9)	

*AFP* alpha-fetoprotein. *Data were missed in some cases; we figured out the number beside each parameter*

Patients with atypical HCC radiological findings were young (65.4%, < 60 years) and had no history of viral etiology in 13.5% of cases (*p* < 0.001 and *p* = 0.03, respectively). The pathological assessment revealed that 23.9% of cases were poorly differentiated (*p* = 0.02). In addition, the clear cell and neutrophil-rich subtypes were significantly associated with atypical features (18.2% and 3.5%, respectively) (*p* < 0.001). Similarly, the presence of dominant intra-tumoral fibrosis (2.8%) was significantly associated with the presence of atypical features (*p* = 0.01). On the other hand, pathognomonic bile formation and necrosis were easily identified in the surgical specimens as compared with liver biopsy (*p* < 0.001, for both). The absence of liver cirrhosis (32.5%) was significantly observed in the atypical cases (*p* = 0.03).

### Characteristic clinicopathological features of the several HCC variants

Table 5 summarized the clinicopathological characteristics of each variant.

**Table 5:** The characteristic clinicopathological features of the special HCC variants

HCC special variants	WHO edition	Clinical and laboratory findings	Gross findings	Prognostic pathological parameters	OS data and recurrence
**1- Macrotrabecular massive** (9%)	2019	Median age (60 years) Male (78.3%) HCV (96.8 %) DAAs (42.9%) Median AFP (300ng/dl)	Median size (6.00 cm) Multifocal (21.7%) Non-cirrhosis (31.1%)	Poor grade (37%) Late stage (17.4%) LVI (63%)	Recurrence (45.5%) Mean OS ± SD (32.35 ± 5.23)
**2- Clear cell HCC** (6.7 %)	2010–2019	Median age (58 years) Male (79.4%) HCV (90.5%) DAAs (37.5%) Median AFP (28 ng/dl)	Median size (5.00 cm) Multifocal (29.0%) Non-cirrhosis (27.6%)	Poor grade (4 %) Late stage (0 %) LVI (33.3%)	Recurrence (0%) Mean OS ± SD (41.05 ± 7.94)
**3- Steatohepatitic HCC** (2.9 %)	2019	Median age (59 years) Male (86.7%) HCV (91.7 %) DAAs (37.5%) Median AFP (16 ng/dl)	Median size (4.00 cm) Multifocal (26.7%) Non-cirrhosis (20%)	Poor grade (60%) Late stage (7.1%) LVI (42.9%)	Recurrence (16.7%) Mean OS ± SD (78.36 ± 16.52)
**4- Scirrhous HCC** (2.2 %)	2010–2019	Median age (59 years) Male (72.7 %) HCV (88.9%) DAAs (66.7%) Median AFP (30 ng/dl)	Median size (4.00 cm) Multifocal (27.3 %) Non-cirrhosis (27.3%)	Poor grade (42.9%) Late stage (0%) LVI (57.1%)	Recurrence (33.3%) Mean OS ± SD (17.57 ± 4.29)
**5- Fibrolamellar HCC (FLC)** (1.6%)	2010–2019	Median age (23 years) Male (57.1%) HCV (14.3%) DAAs (NA) Median AFP (9 ng/dl)	Median size (8.5 cm) Multifocal (0%) Non-cirrhosis (100%)	Poor grade (NA) Late stage (0%) LVI (33.3%)	Recurrence (66.7%) Mean OS ± SD (72 ± 23)
**6- Sarcomatoid HCC** (2%)	2010	Median age (59 years) Male (90%) HCV (85.7%) DAAs (100%) Median AFP (62 ng/dl)	Median size (6.00 cm) Multifocal (30%) Non-cirrhosis (0%)	Poor grade (100%) Late stage (33.3%) LVI (25%)	Recurrence (33.3%) Mean OS ± SD (36 ± 0.00)
**7- Chromophobe HCC** (0.8%)	2019	Median age (60 years) Male (75%) HCV (100%) DAAs (75%) Median AFP (22.5 ng/dl)	Median size (5.00 cm) Multifocal (0%) Non-cirrhosis (50%)	Poor grade (0%) Late stage (0%) LVI (50%)	Recurrence (25 %) Mean OS ± SD (29.67 ± 5.99)
**8- LEL-HCC** (0.2%)	2010–2019	Median age (42 years) Male (100%) HCV (100%) DAAs (0%) Median AFP (2 ng/dl)	Median size (2.00 cm) Multifocal (0 %) Non-cirrhosis (100 %)	Poor grade (100%) Late stage (0%) LVI (0%)	Recurrence (0 %) Mean OS ± SD (60 ± 0.00)
**9- Neutrophils rich HCC** (1%)	2019	Median age (60 years) Male (80 %) HCV (80 %) DAAs (33.3 %) Median AFP (22.5 ng/dl)	Median size (8.00 cm) Multifocal (40%) Non-cirrhosis (0%)	Poor grade (80 %) Late stage (NA) LVI (100 %)	Recurrence (50%) Mean OS ± SD (6.5 ± 3.89)
**10- cHCC-CC** (0.6%)	2010–2019	Median age (59 years) Male (100%) HCV (100%) DAAs (0%) Median AFP (4000 ng/dl)	Median size (8.00 cm) Multifocal (33.3%) Non-cirrhosis (0%)	Poor grade (NA) Late stage (100%) LVI (66.7%)	Recurrence (NA) Mean OS ± SD (1 ± 0.00)

*HCC* hepatocellular carcinoma, *WHO* World Health Organization, *HCV* hepatitis C virus, *OS* overall survival, *DAAs* direct acting anti-viral, *AFP* alpha-fetoprotein, *LEL-HH* lymphoepithelioma like HCC, *LVI* lymphovascular invasion, *c-HCC-CC* combined hepatocellular-cholangiocarcinoma

### Macrotrabecular massive HCC

**Fig. 5:**
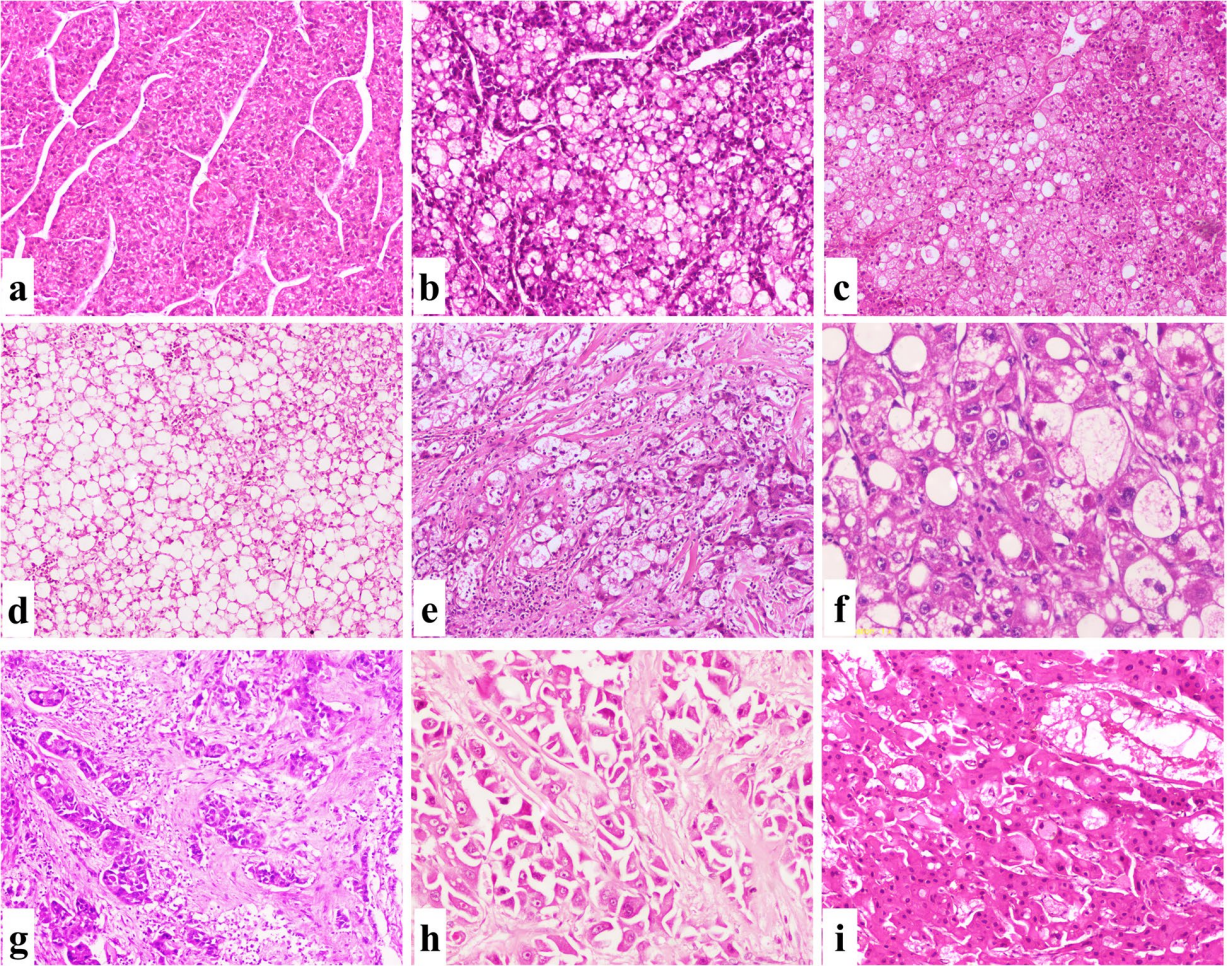
Microscopic aspects of special HCC variants. **a** A macrotrabecular massive subtype showed trabeculae > 10 cells in thickness (IHC, 100×). **b** A clear cell subtype showed sheets of hepatocytes contained high glycogen and lipid content (IHC, 100×). **c** A clear cell subtype showed focal fatty changes (IHC, 100×). **d** A clear cell subtype showed severe fatty changes (IHC, 100×). **e** A steatohepatitic subtype showed a triad of fatty change, intra-tumoral fibrosis, and inflammation (IHC, 100×). **f** A steatohepatitic subtype showed Mallory hyaline bodies (IHC, 200×). **g** A scirrhous subtype showed compressed cords of hepatocytes within desmoplastic stroma (IHC, 100×). **h** A FLC variant showed hepatocytes with abundant eosinophilic cytoplasm, prominent eosinophilic nucleoli separated by lamellated collagen bundles (IHC, 100×). **i** A FLC showed intra-tumoral pale bodies (IHC, 200×)

This variant is defined as > 50% of the tumor showing a trabecula of ≥ 10 cell thickness. Macrotrabecular massive HCC was associated with a high serum AFP level, large tumor size, and frequent LVI. Tumor recurrence was reported in 45.5% of cases and was linked to a short OS (Fig. 5a, b).

### Clear cell HCC

This variant is defined as > 80% of tumor cells being clear cells. Despite one-third of cases being multicentric, all cases were diagnosed at an early stage with no tumor recurrence. Clear cell HCC was associated with the third-longest OS. The background liver shows no prominent fatty changes (Fig. 5c, d).

### Steatohepatitic HCC

This variant is considered a malignant mimicker of steatohepatitis. In the present study, most cases were related to HCV. The characteristic features of steatohepatitic HCC include median tumor steatosis (50%), tumor-infiltrating lymphocytes (TILs) (20%), and intra-tumoral fibrosis (10%). A poor grade was noted in two-thirds of cases with frequent LVI. However, tumor recurrence was rarely reported, and the OS of patients with this variant was the longest. The background liver showed no prominent inflammatory or fatty changes (Fig. 5e, f).

### Scirrhous HCC

This variant is defined as having a dense desmoplastic stroma of > 50% that compresses the malignant hepatocytes in a cord-like pattern. More than half of the cases showed LVI, with a third having tumor recurrence. This variant was associated with a short OS (Fig. 5g).

### FLC

This variant has a characteristic pathological triad of large eosinophilic cells with large vesicular nuclei and prominent eosinophilic nucleoli and is separated by parallel arrays of dense fibrous septa. This variant characteristically occurs at a young age without a viral etiology and a non-cirrhotic liver. Almost all cases had a large tumor size, with one-third showing LVI. Tumor recurrence was reported in two-thirds of cases, and this variant was associated with a long OS (Fig. 5h, i).

### Sarcomatoid HCC

**Fig. 6:**
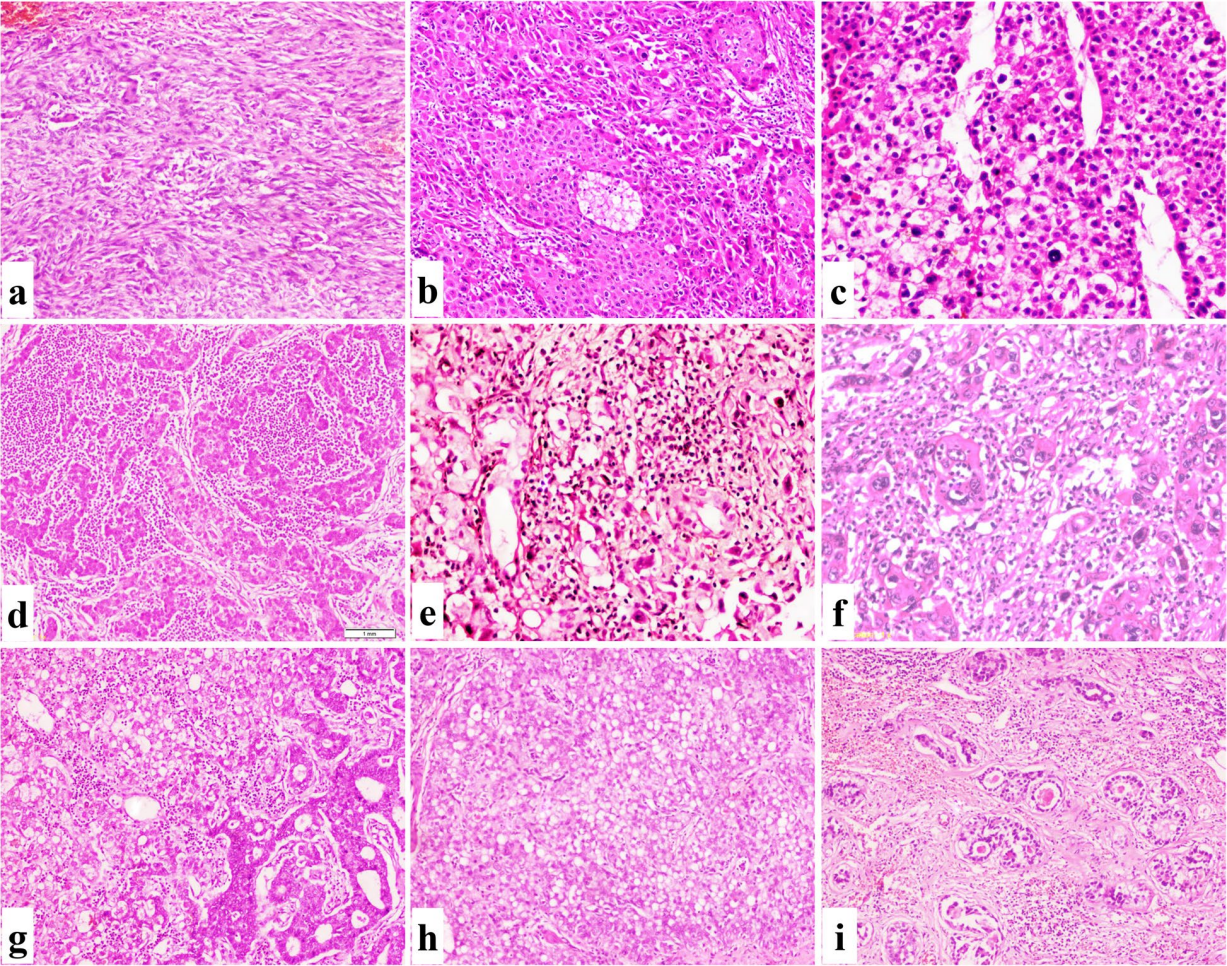
Microscopic aspects of special HCC variants, continued. **a** A sarcomatoid subtype showed spindle tumor cells arranged in fascicular pattern (IHC, 100×). **b** A chromophobe subtype showed sheets of tumor cells with clear to eosinophilic cytoplasm, sharp cell border, and paranuclear halos (IHC, 100×). **c** A chromophobe subtype showed bland nuclei with area of abrupt anaplasia (IHC, 200×). **d** A LEL-HCC subtype showed intra-tumoral lymphocytes outnumbered the tumor cells (IHC, 100×). **e**–**f** A neutrophil-rich subtype showed numerus and diffuse neutrophils within the tumor (IHC, 200×). **g** A c-HCC-CC showed mixed hepatocytic and cholangiocytic areas of differentiation (IHC, 100×). **h** c-HCC-CC showed the hepatocytic differentiation area (IHC, 100×). **i** c-HCC-CC showed the cholangiocytic differentiation area (IHC, 100×)

This variant is defined as classic HCC with various malignant spindle cells and is characterized by a large tumor size and prominent tumor necrosis. A third of the cases were diagnosed at a later stage and exhibited tumor recurrence. The median OS of this variant was 3 years (Fig. 6a).

### Chromophobe HCC

This variant is defined as HCC with light clear cytoplasm and bland tumor nuclei; however, an area of frank anaplasia could be present. Half of the cases had a large tumor size with a positive LVI. Tumor recurrence was reported in 25% of cases, with a mean ± standard deviation of 29.67 ± 5.99 (Fig. 6b, c).

### Lymphoepithelioma-like carcinoma (LEL-HCC)

This variant occurs when the intra-tumoral lymphocytes, with a characteristic dense eosinophilic cytoplasm, outnumber the tumor cells and lack the area of classic HCC. Only one case was reported in our institute, a 42-year-old male who was positive for HCV [14]. A follow-up of the patient revealed that he has been recurrence-free for the past 60 months (Fig. 6d).

### Neutrophil-rich HCC

This variant has numerous intra-tumoral neutrophils obscuring tumor cells in a viable tumor area (not related to necrosis). It is characterized by a large tumor size, poor tumor grade, and frequent LVI. Tumor recurrence was reported in half of the cases, and patients had a short OS (Fig. 6e, f).

### HCC combined with cholangiocarcinoma (cHCC-CC)

This variant is defined as the unequivocal presence of both HCC and cholangiocarcinoma within the same tumor, either in proximity to each other or deeply intermingled. Cases with this variant had high serum AFP levels, large tumor sizes, and frequent LVI and were diagnosed at later stages. All cases were associated with LN metastasis, and the OS was one month (Fig. 6g, i).

### Recurrence and survival data

HCC recurrence was reported in 55/173 (31.8%) of cases, and the recurrence site was primarily in the liver (53/55, 96.4%), followed by the bones and LNs. Time to recurrence ranged from 2 to 74 months.

Regarding HCC survival, 49.5% died from HCC. The mean OS was 26.89 ± 21.102 months, with a median of 24 months.

**Fig. 7:**
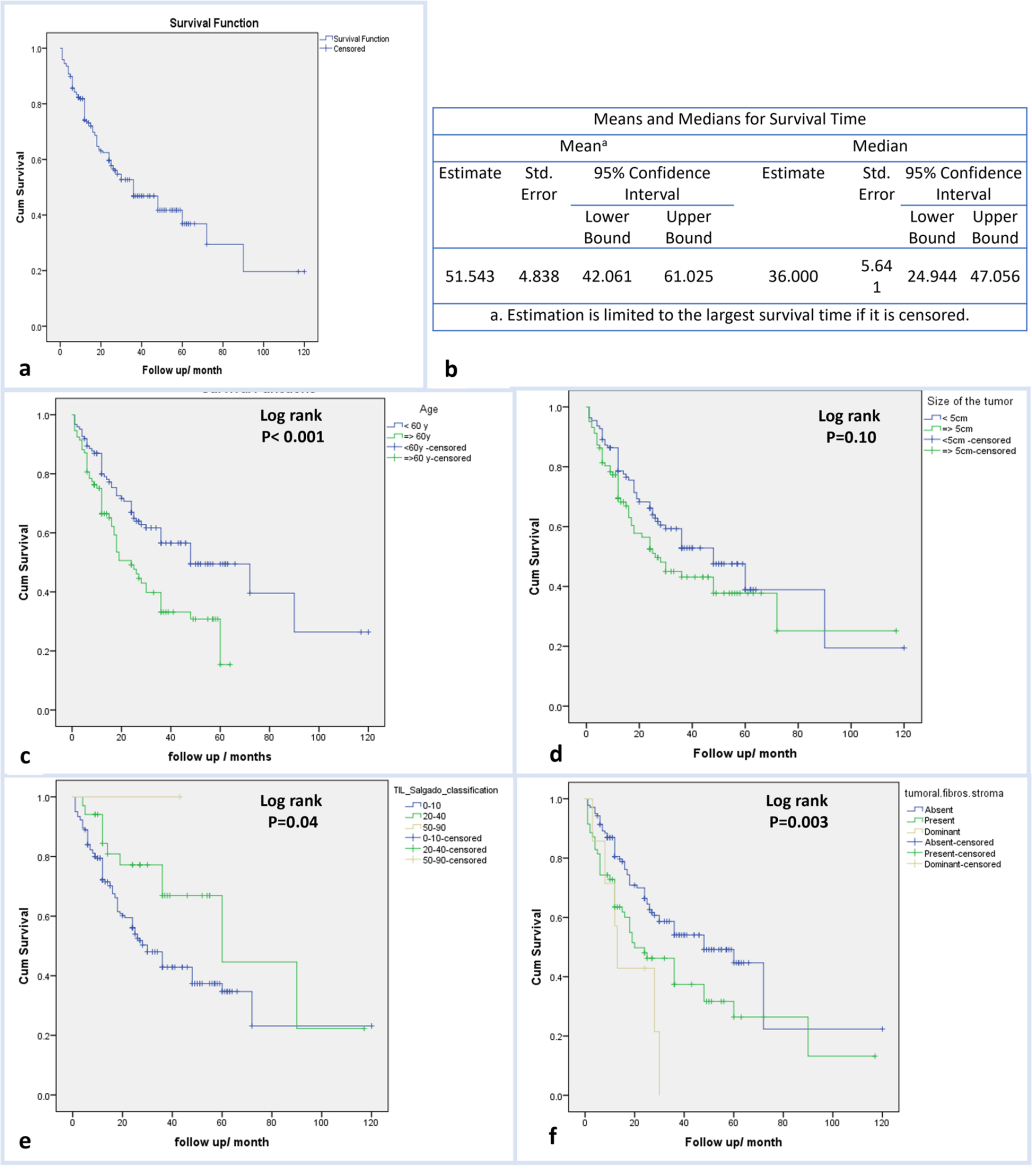
Survival data of HCC patients. **a** Kaplan-Meier survival curve demonstrating the OS of HCC patients. **b** Table demonstrated the mean and median survival time for HCC patients. **c** Kaplan-Meier survival curve demonstrating the impact of patients’ age on the OS. **d** Kaplan-Meier survival curve demonstrating the impact of tumor size on the OS. **e** Kaplan-Meier survival curve demonstrating the impact of TILs on the OS. **f** Kaplan-Meier survival curve demonstrating the impact of intra-tumoral fibrous stroma on the OS

Univariate and multivariate Cox proportional hazards regression were performed to screen prognostic factors for OS, which revealed that older age (*p* = 0.01), high TILs (*p* = 0.006), tumor size (*p* = 0.007), and severe intra-tumoral fibrous stroma (*p* = 0.02) were independent prognostic factors for the OS of patients with HCC (Table 6, Fig. 7).

**Table 6:** The univariate and multivariate Cox proportional hazard regression for screening prognostic factors for HCC overall survival

Studied variable	Univariate		Multivariate	
	***p*** value	HR (95% CI)	***p*** value	HR (95% CI)
**Older age**	0.01	1.03 (1.02–1.05)	0.01	1.03 (1.01–1.06)
**Sex** (male)	0.18	0.6 (0.4–1.18)		
**Viral etiology**	0.94	0.9 (0.4– 2.1)		
**Prior HCV treatment**				
IFN	0.57	1.2 (0.6–2.0)		
DAAs	0.28	0.7 (0.3–1.3)		
**Serum AFP**	0.64	1.0 (1.0–1.0)		
**Tumor focality** (multiple)	0.21	1.3 (0.8–1.9)		
**Tumor size**	0.01	1.1 (1.017–1.2)	0.007	1.1 (1.02–1.2)
**Pathological grade**				
I	Reference			
II	0.91	0.9 (0.4–1.8)		
III	0.46	1.3 (0.6–2.8)		
**Tumor TIL Salgado**				
0–10	Reference			
20–40	0.03	0.4 (0.2–0.9)	0.006	0.3 (0.2–0.7)
**Intra-tumoral fibrous stroma**				
Absent	Reference			
Present	0.01	1.6 (1.1–2.4)	0.002	1.9 (1.2–2.8)
Dominant	0.01	2.9 (1.2–6.8)	0.02	2.8 (1.2-6.6)
**Pathological stage**				
1	Reference			
2	0.66	1.3 (0.3–5.7)		
3	0.53	1.6 (0.3–7.2)		
4	0.30	2.5 (0.4–15.5)		
**Tumor stage** (late)	0.25	1.4 (0.7–2.5)		
**Liver cirrhosis**	0.22	1.2 (0.8–1.9)		
**LVI**	0.27	1.2 (0.8–2.1)		

*HCC* hepatocellular carcinoma, *HCV* hepatitis C virus, *IFN* interferon, *DAAs* direct acting anti-viral, *AFP* alpha-fetoprotein, *TILs* tumor-infiltrating lymphocytes, *LVI* lymphovascular invasion, *HR* hazard ratio

## Discussion

HCC remains a major health problem despite the emergence of different preventive and treatment modalities [15]. The mean onset of HCC in our study was during the sixth decade, which is compatible with our previous data [2]. Similar data on the age at the time of HCC diagnosis was reported in the United States, Europe, Canada, and Japan. All these countries share a common HCV etiology [16]. However, patients of African origin with HCC have a younger age and are associated with an HBV infection, aflatoxin, and p53 mutations. This signature is not related to liver cirrhosis, alcohol intake, or smoking [17]. Therefore, the variation in age could be attributed to different causative factors, such as race, ethnicity, and genetic susceptibility [18]. In addition, HCV infection is usually acquired in adulthood compared with HBV infection, which delays the onset of HCC development [19]. This is in line with our results that patients with nonviral-related HCC were significantly younger than patients with HCV-related HCC.

The present study showed a fivefold increase of HCC incidence in males compared with females. However, the male/female ratio was higher in young patients with HCC (< 40 years) than in older patients (8 vs. 5, respectively), but this was not statistically significant. Male predominance in HCC was related to increased exposure to risk factors and androgens (AR) and estrogens (ER) [19]. ER protects against HCC development through the modulation of inflammation and induction of apoptosis [20]. Contrarily, AR overexpression promotes HCC initiation and aggressiveness [21, 22]. The current study revealed a significant association between male gender and tumor multifocality and advanced pathological stage, which supported the poor prognostic impact of being male.

The serum AFP level is still an important diagnostic biomarker for HCC diagnosis; however, the optimal AFP cutoff point is controversial. Serum AFP could be elevated in other tumors or chronic liver disease and cirrhosis [23, 24]. In the current study, a serum AFP level of > 20 ng/dL was detected in 60% of the cases. Zhang et al. reported that a serum AFP of 400 ng/dL has the best sensitivity and specificity when diagnosing HCC. However, the threshold of 20 ng/dL should be considered during HCC surveillance programs [25]. Furthermore, the cases with the highest serum AFP levels (> 400 ng/dL) in the current study was significantly associated with poor HCC prognostic parameters, large tumor sizes, late stage, and early HCC recurrence. The poor prognosis of cases with elevated serum AFP levels could result from the role of AFP in inducing HCC cell proliferation by activating the cAMP-PKA signal transduction pathway and altering the K-ras gene expression [26]. In addition, a high AFP level induces HCC angiogenesis through the activation of vascular endothelial growth factor expression [27]. Therefore, our study postulated a screening and investigation of patients with HCV with serum AFP levels of > 20 ng/dL. Furthermore, serum AFP levels of > 400 ng/dL was considered to indicate a poor HCC prognosis and high recurrence.

The present study showed that HCV was the dominant etiology of HCC in Egypt, and it remains the leading cause of HCC in Europe, North America, and Japan [7]. Despite the emergence of DAAs, data on the risk of HCC post-DAA use is not well established. DAA-induced SVR mediated immunological changes and induced HBV reactivation, which did not impact HCC incidence [28]. Conti et al. assumed that the high HCC risk post-DAAs was related to the severity of liver cirrhosis and previous HCC history rather than an HCV genotype or DAA regimen [29]. In addition, patients treated with DAAs were elderly patients with cirrhosis with high serum AFP levels, which are the main risk factors for HCC development [30]. In contrast, Gitto et al. reported a reduced risk of HCC in patients who achieved SVR post-DAA use [31]. Similarly, Kilany et al. found a low incidence of HCC in patients with HCV treated with DAA with advanced fibrosis and cirrhosis. The factors associated with a high risk of HCC were decompensated cirrhosis, metabolic syndrome, failure to obtain SVR, and a baseline AFP level of ≥ 10 ng/dL [32]. In the current study, post-IFN HCC tended to be solitary tumors, while tumor necrosis was significantly observed in post-DAA HCC. The aggressive behavior of post-DAA HCC has been previously reported. Post-DAA HCC tended to be large multifocal infiltrative tumors with frequent LVI and LN invasion [33, 34]. Further studies could explain the pathogenic and prognostic characteristics of this tumor to better understand the molecular pathways of post-DAA HCC development.

The background liver plays an important role in the development and progression of HCC. Liver cirrhosis is an independent risk factor for HCC, specifically in patients with HCV [15]. However, 25% of the HCC cases in the current study occurred even in non-cirrhotic livers in older patients and were characterized as large and multifocal with frequent LVI and even bile duct invasion. Older age at onset and the aggressive course of non-cirrhotic HCC could be attributed to the lack of symptoms and maintained hepatic function, which interfere with early diagnosis [35, 36]. This delay in diagnosis contributes to the advanced stage of the disease and the high rate of extrahepatic metastasis on presentation of patients [37]. The lack of significant etiological differences between cirrhotic and non-cirrhotic HCC in the present study could indicate the complexity and different pathogenic pathways of this subtype.

The role of imaging, namely, triphasic CE-CT and dynamic MRI, permits a definitive diagnosis of HCC in high-risk patients without the need for invasive pathological confirmation [38]. The Liver Imaging Reporting and Data System (LI-RADS) aids in the accurate stratification of HCC, including the small-sized ones [39]. Our study revealed that young patients had negative viral etiology and absent cirrhotic changes on liver biopsy. This category of patients is not considered high risk and are not eligible for LI-RADS scoring [40]. HCC cases with atypical imaging results in the current study showed characteristic poor pathological grades, frequent clear cell changes, intra-tumoral fibrosis, and intra-tumoral neutrophils. Although radiological imaging is nonspecific for most HCC subtypes raising on liver cirrhosis, it may represent a diagnostic challenge for radiologists in 40% of cases [41]. Sarcomatoid and scirrhous HCCs appeared as hypovascular tumors with rim-like enhancements and targetoid patterns on radiological images, respectively, which indicate pathological confirmation [41]. Liu et al. found no significant radiological differences between clear cell and classic HCC. However, five clear cell HCC cases exhibited atypical triphasic CT features: three exhibited gradual contrast enhancement during the portal phase and two showed minimal enhancement with maintained hypoattenuation at the arterial and venous phases [42]. Therefore, awareness of the special variants of HCC may have potential clinical implications for the patients’ prognosis and may serve as a diagnostic clue for the atypical imaging findings [43].

Large-scale genomic analyses have identified the key cell signals, mutational landscapes, and metabolic derangements related to hepatocarcinogenesis. This results in molecular subclasses with increasing evidence for morpho-molecular correlation [10, 44]. In the current study, macrotrabecular massive subtype, scirrhous, sarcomatoid, neutrophil-rich, and cHCC-CC represent special types of HCC with poor prognosis and survival [12, 45–47]. On the other hand, clear cell, steatohepatitic, FLC, LEL-HCC, and chromophobe HCCs were associated with good prognostic parameters [10, 48, 49]. Although the data were not statistically significant (due to the small number of some variants), they could highlight the importance of the morphological classification of HCC subtypes. The morpho-molecular classification may indicate a unique gene expression signature that may help in the prognostication and therapeutic management of patients [44].

The difference in the frequency of some HCC variants could have resulted from the lack of definite criteria, such as the thickness of the trabeculae in macrotrabecular massive variant or the wide range for cutoff points in the clear cell, scirrhous, and steatohepatitic subtypes [12, 45]. In addition, the low frequency of the steatohepatitic variant in the present study (2.9% versus 5–20% in the previous studies) with no significant steatohepatitis in the background liver could be linked to the low incidence of nonalcoholic fatty liver disease-related HCC. However, the association between steatohepatitic HCC and metabolic syndrome is not yet well elucidated [50]. Similarly, chromophobe HCC was rare, occurred in males, had a poor grade and frequent LVI, and had similar OS as classic HCC. The poor grade could be attributed to the abrupt anaplasia frequently reported in this subtype [51]. Kang et al. reported a higher frequency of chromophobe HCC with female predominance [52]. Future studies are recommended for the clinical characteristics of chromophobe HCC.

Although some HCC special variants shared similar histopathological findings, they had diverse prognostic outcomes. Scirrhous HCC and FLC both had characteristic fibrous bundles, but they differed in the nature of the fibrous stroma: dense lamellated collagenous bands in FLC versus abundant fibroblasts and stemness-related markers in scirrhous HCC [53]. In addition, scirrhous HCC may share some of the CC gene expression profile and activated epithelial-mesenchymal transition pathway, which influence aggressive behavior [46]. Similarly, LEL-HCC and neutrophil-rich HCC showed opposite clinical, prognostic, and survival data. Neutrophils are the first immune cells that enter the tumor microenvironment and promote tumor growth and metastasis, while tumor lymphocytes are engaged in the host-mediated immune response against tumor cells [54, 55]. Therefore, peripheral neutrophil and lymphocyte counts could be independent noninvasive predictors of HCC prognosis [56]. Therapeutic targeting of different immune cells could be beneficial in special HCC subtypes.

cHCC-CC is a rare and aggressive tumor representing a distinct type of primary liver cancer originating from hepatic stem cells [57]. Current evidence suggests that cHCC-CC shares some of the clinical, etiological, and genetic backgrounds of both HCC and CC, resulting in an intermediate prognostic course [58].

With a median OS of 2 years, the survival data of patients with HCC in this study was compatible with previous studies [59]. Older age, large tumor size, and dense intra-tumoral fibrous stroma indicating scirrhous HCC were the independent prognostic factors affecting short-term survival. This is in agreement with several studies that illustrated that tumor size is an independent prognostic for HCC survival [59, 60]. In a meta-analysis conducted by Ding et al., TILs played a prognostic role in the postsurgical resection HCC [61].

## Conclusions

HCC is a heterogenous tumor with diverse clinical, pathological, and prognostic parameters. Age, male gender, elevated serum AFP level, tumor size, background liver, and special histopathological variants are considered key indicators for patient outcomes. Post-DAA HCC could have an aggressive behavior compared with their post-IFN counterparts. The morphological classifications of HCC could serve as diagnostic clues for atypical radiological imaging findings, which can help in predicting the prognosis of patients and personalizing treatment.

## Data Availability

The datasets are available from the corresponding author on reasonable request.

## Abbreviations

AFP: Alpha-fetoprotein
AJCC: American Joint Committee of Cancer
AR: Androgens
CE-CT: Contrast-enhanced triphasic computed tomography
DAAs: Direct-acting antiviral agents
ER: Estrogens
FLC: Fibrolamellar carcinoma
HBV: Hepatitis B virus
HCC: Hepatocellular carcinoma
HCV: Hepatitis C virus
IFN: Interferon
LEL-HCC: Lymphoepithelioma-like carcinoma
LI-RADS: Liver Imaging Reporting, and Data System
LN: Lymph node
LVI: Lymphovascular invasion
MRI: Magnetic resonance imaging
OS: Overall survival
SVR: Sustained virological response
TILs: Tumor-infiltrating lymphocytes
WHO: World Health Organization
